# New oncology trial designs and equipoise: Commentary on Heynemann et al.

**DOI:** 10.1177/17407745261471455

**Published:** 2026-09-11

**Authors:** Jonathan Kimmelman

**Affiliations:** 1 Department of Equity, Ethics and Policy, School of Population and Global Health, McGill University, Montreal, QC, Canada

**Keywords:** equipoise, oncology, clinical trials, seamless trials, adaptive trials, platform trials

## Abstract

This essay responds to Heymemann et al.’s analysis of the applicability of clinical equipoise to emerging trial methods in cancer. Briefly, I argue that equipoise presses study designers to conduct trials at the point of maximum community uncertainty. Accordingly, equipoise encourages maximum information gain per patient enrolled (or inversel, minimal patient burden per gain in information). As such, equipoise is as much a condition for the ethical conduct of new study designs as it is for old ones. I close by suggesting that, whereas Heymemann et al. suggest new trial designs present a challenge to the operationalization of equipoise, I argue that equipoise presents a challenge for those wishing to operationalize new trial designs.

Typical clinical trials have a beginning and an end. They test one primary hypothesis. Analysis and reporting are mostly held in abeyance until completion, whereupon the statistician views the results. Increasingly, however, trials are employing much more complex and dynamic approaches. They often test multiple primary hypotheses. They lack defined terminals. Analyses are conducted to adapt the trial itself.

Heynemann et al.^1^ argue thoughtfully that such newer trials strain the relevance and implementation of a foundational moral principle for clinical trials: clinical equipoise. Roughly, this principle holds that ethical trial conduct is conditional on uncertainty within expert communities as to the clinical merits of a new treatment in comparison with the standard of care, and the trial being likely to resolve this community uncertainty. In Heynemann et al.’s analysis, clinical equipoise becomes just one moral instrument in a “toolbox” of others that investigators and ethics committees use when evaluating adaptive trials. Three referees – each steeped in interpretations of clinical equipoise – found their argument plausible. However, I think our referees may have been overly generous.

There are as many specifications of clinical equipoise as there are commentators.^2–5^ My own approach is indebted to them and grounds a more generic equipoise (i.e. not necessarily *clinical* equipoise) on “moral efficiency,” which can be understood here as getting the most amount of useful information per patient enrolled in a trial.

Imagine you have an expert medical community, and they are presented with a decision about an action, like further investment in a new drug. Perhaps this action is regulatory approval. The decision is a technical one. It involves the synthesis, interpretation, and evaluation of evidence. The decision is also ethically weighty. Further investing in the drug will expose more persons with unmet health needs to risks and burdens of a new drug; it will also commit scarce resources. A decision not to further invest in the drug is also weighty, insofar as it runs up opportunity cost for science and patients should the drug be effective. The moral warrant for making this decision rests on evidence. In particular, it rests on informed expert consensus that investing is (or is not) the right thing to do, given what we know and given a socially acceptable tolerance for risk. When action-guiding hypotheses aim at directly informing care decisions, this equipoise takes a specific form – the version originally formulated by Benjy Freedman:^6^ *clinical* equipoise. In this instance, the uncertainty concerns how best to medically treat a population.

Equipoise furnishes the moral license for using patients to resolve expert uncertainty. It requires first that, at the outset of the trial, experts have divided or conflicting opinions about an action-guiding hypothesis. It second requires that experts agree that, if the trial is well run, it is likely to extinguish community uncertainty and coalesce expert consensus needed for morally justified action. As observed by Ben Djulbegovic,^4^ by withholding trial initiation until there is a roughly even split in expert opinion, equipoise helps maximize a community’s information gain per patient enrolled in a trial. It also maximizes information gain per patient enrolled by ending the trial once consensus has solidified. By treating science and ethics as constituting each other, equipoise promotes moral efficiency.

Heymemann et al. discuss several methodological innovations that seem to challenge equipoise. I will focus my remarks on one, seamless phase 2/3 trials, though my arguments extend to the other two. They argue that drug administration in the phase 2 portion for many seamless phase 2/3 trials starts out of clinical equipoise (I agree with them,^7,8^ though the first condition of clinical equipoise is met insofar that no patient is withheld from the standard of care) and only later comes to meet the conditions of clinical equipoise. Ergo, clinical equipoise cannot furnish a comprehensive moral framework for such trials. It requires supplementation with other moral frameworks from their toolbox.

But let’s think this through, using a hypothetical seamless phase 2/3 trial where two doses are tested against a historical comparator in the phase 2 portion, and the best-performing dose is advanced into randomized phase 3 testing (let’s also assume data from the phase 2 portion will be pooled with those from phase 3). The phase 2 portion begins in a state of equipoise (but not clinical equipoise). That is, some experts think the two doses are promising enough to develop further, some experts think the drug will fizzle, and almost no one thinks the drug is ready for clinical application. The trial designers have established a threshold of promise – based on safety and an effect on a surrogate outcome – to transition the better-performing dose into phase 3 testing. A data monitoring committee will oversee the crossing of this threshold. One dose perturbs the equipoise grounding phase 2, and in so doing – experts update their beliefs and another equipoise is pulled into being: *clinical* equipoise. Now, some experts – looking at all the phase 2 results and other forms of evidence – think the new drug will prove better than what is used in clinical care. Others are doubtful. The phase 3 portion has been designed to convert the skeptics into proponents (or the reverse).

These different equipoises have implications for consent. During the phase 2 portion, the participants are told that risks and burdens are likely, but major benefits are not – and that the trial is being run to determine if the drug is worth further development. They are also told that if the drug turns out to be promising enough for further development, the trial will switch to testing whether it is better than the standard of care. During the phase 3 portion of the trial, patients are told the new treatment is believed to be competitive with the standard of care they would receive outside the study – it could be better, or it could be worse than what they would otherwise receive. Those patients who transition from the phase 2 to phase 3 portions are reconsented: experts have enough evidence to think the drug and dose they were receiving could be better than the standard of care (but not necessarily – it could be worse); would they like to continue enrollment? Such reconsenting when thresholds are crossed may erode certain operational efficiencies for seamless designs, but that is the price we pay to respect participants.

Heynemann et al. imply – I think – emerging trial techniques demand adaptation of ethical principles. What I am suggesting is that ethics should be embedded in the trial itself. Uncertainty concerning an actionable clinical hypothesis grounds the ethics of trial enrollment. The extinction of uncertainty grounds the planned decision and transition criteria. If our task is to maximize moral efficiency, we begin investigations with maximum uncertainty, we use good moral thinking to pre-specify thresholds of evidence for action, and we use the fewest patients possible – at least burden – to decide whether to advance a strategy to further testing (or practice) or whether to revise our approach. And we reconsent patients once significant decision criteria are met.

In a single concept, equipoise joins norms of evidence and belief change that are accepted in professional communities of experts with norms concerning the moral conduct of medicine (and/or human research). A common critique of clinical equipoise, implicit in Heynemann et al., is that clinical equipoise defies straightforward operationalization: what to do when p-values creep toward significance. I think this critique is easy to dismiss. All moral standards – the notion of requiring “valid” informed consent or “favorable” risk/benefit – defy rote operationalization. Show me a moral principle whose application to a decision doesn’t entail mindful judgment, and I will show you a decision that can be made without appeal to a moral principle.

Specifying the application of equipoise to newer trial designs takes some thought. Heynemann et al. press us to begin that thinking. But the bottom line is this: equipoise (and clinical equipoise) pose a challenge to those designing and implementing new trial designs in oncology, not the other way around. This challenge entails designing, conducting, and reporting trials in ways that minimize each patient’s burden while maximizing the extent to which medical communities change their beliefs and make good decisions.
